# Supplementary material for: High-dose testosterone supplementation disturbs liver pro-oxidant/antioxidant balance and function in adolescent male Wistar rats undergoing moderate-intensity endurance training
Source: PeerJ. 2020 Nov 19;8:e10228. doi: 10.7717/peerj.10228 (PMC7680624; doi:10.7717/peerj.10228)
Supplement: Supplemental Information 4 [file peerj-08-10228-s004.docx]

# 1. [M. Barroso](https://www.muscleandfitness.com/author/mark-barroso/). 8 tips for balancing bodybuilding and endurance training.

# Available at <https://www.muscleandfitness.com/workouts/workout-tips/8-tips-balancing-bodybuilding-and-endurance-training>

# Accessed September 23, 2020

2. Anabolic-androgenic steroid stacking regimens

Available at <http://www.steroid.com/Steroid-Stacking.php>

and <http://www.anabolics.com/pages/Steroid-Stacking#WJzYZvnhCkw>

Accessed September 23, 2020
